# Comparison of epidemiology and clinical characteristics of infections by human parechovirus vs. those by enterovirus during the first month of life

**DOI:** 10.1007/s00431-015-2566-9

**Published:** 2015-05-16

**Authors:** María Cabrerizo, Gloria Trallero, María José Pena, Amaia Cilla, Gregoria Megias, Carmen Muñoz-Almagro, Eva Del Amo, Diana Roda, Ana Isabel Mensalvas, Antonio Moreno-Docón, Juan García-Costa, Nuria Rabella, Manuel Omeñaca, María Pilar Romero, Sara Sanbonmatsu-Gámez, Mercedes Pérez-Ruiz, María José Santos-Muñoz, Cristina Calvo, *And the study group of “Enterovirus and parechovirus infections in children under 3 years-old, Spain” PI12-00904

**Affiliations:** Enterovirus Unit, National Centre for Microbiology, Health Institute “Carlos III”, Madrid, Spain; Hospital Negrín de Gran Canaria, Las Palmas, Spain; Pediatrics Department, Hospital Severo Ochoa, Avda. Orellana, s.n., Leganés, 28911 Madrid Spain; Hospital de Burgos, Burgos, Spain; Hospital San Joan de Deu, Barcelona, Spain; Hospital Virgen de la Arrixaca, Murcia, Spain; Hospital de Ourense, Ourense, Spain; Hospital Santa Creu i Sant Pau, Barcelona, Spain; Hospital Miguel Servet, Zaragoza, Spain; Hospital La Paz, Madrid, Spain; Hospital Virgen de las Nieves, Granada, Spain

**Keywords:** Enterovirus, Human parechovirus, Sepsis, Fever, Neonate

## Abstract

Human parechoviruses (HPeV) have been recently recognized as important viral agents in paediatric infections. The aims of this study were to investigate the HPeV infection prevalence in infants <1 month in Spain and, secondly, to analyse the clinical and epidemiological characteristics of the infected patients compared with those infected by enterovirus (EV). Infants <1 month with neurological or systemic symptoms were included in a multicentre prospective study. EV and HPeV detection by RT-PCR and genotyping were performed in cerebrospinal fluids (CSF), sera or throat swabs. Out of the total of 84 infants studied during 2013, 32 were EV positive (38 %) and 9 HPeV positive (11 %). HPeV-3 was identified in eight cases and HPeV-5 in one. Mean age of HPeV-positive patients was 18 days. Diagnoses were fever without source (FWS) (67 %), clinical sepsis (22 %) and encephalitis (11 %). Leukocytes in blood and CSF were normal. Pleocytosis (*p* = 0.03) and meningitis (*p* = 0.001) were significantly more frequent in patients with EV infections than with HPeV.

*Conclusions*: Although HPeV-3 infections were detected less frequently than EV, they still account for approximately 10 % of the cases analysed in infants younger than 1 month. HPeV-3 was mainly associated with FWS and without leukocytosis and pleocytosis in CSF. In these cases, HPeV screening is desirable to identify the aetiologic agent and prevent unnecessary treatment and prolonged hospitalization.

**Table:** 

**What is Known**: • *Human parechovirus may be a cause of fever and clinical sepsis in the neonatal period*. • *HPeV-3 might be one of the main agents causing severe neonatal neurological infections*.
**What is New:** • *This is the first multicenter prospective study focused on newborns and contributes to a better knowledge of these viral infections. Clinical characteristics of enterovirus and parechovirus infections are compared specifically in the neonatal period*. • *Knowledge of HPeV infections by paediatricians and neonatologists can guide the diagnosis of these patients and avoid unnecessary treatment and prolonged hospitalization*.

## Introduction

Human parechoviruses (HPeV) are RNA viruses belonging to the family of *Picornaviridae*. Formerly described as echovirus 22 and 23 in the *Enterovirus* genus, HPeV were reclassified into their own genus, *Parechovirus*, in the 1990s based on genetic differences and biological properties. Then, they were renamed as HPeV-1 and HPeV-2, respectively [13, 25]. Recently, additional types of HPeV have been reported, and a total of 16 different types have been recognized to date (HPeV-1 to 16). The most common genotype detected worldwide is HPeV-1 followed by HPeV-3. Other types such as HPeV-2 and HPeV-4 are less common.

Infections with HPeV are prevalent in young children and have been associated with mild diseases of the respiratory and gastrointestinal tract but also with serious diseases such as meningitis, encephalitis and sepsis in young infants [3, 12, 13]. According to recent studies, HPeV-3 might be one of the main agents causing severe neonatal neurological infections in Europe [5, 14, 17], although its real incidence is unknown since HPeV detection is not routinely performed in the majority of clinical microbiology laboratories. Clinical data specific for neonates are also scarce.

This study describes the results obtained about the prevalence of HPeV infections in children up to 1 month of age in Spain and the clinical and epidemiological characteristics of the infected patients over a 1-year period. Furthermore, HPeV and enterovirus (EV) infections were compared.

## Methods

### Patients and samples

In 2013, a prospective study was conducted in collaboration with 10 Spanish hospitals. The project was supported with a grant from the Health System (AES; PI12-00904). The personal data of the patients were protected, and the study was approved by the Ethics Committee of the Health Institute Carlos III. Criteria for inclusion were infants <1 month of age with fever without source (FWS), clinical sepsis disease or meningitis/encephalitis admitted to the participant centres, whose parents agreed to sign the informed consent. During the hospital stay, a physician filled out a study questionnaire with the clinical characteristics of the patients. *FWS* was defined as axillary temperature greater than 37.9 °C, which after an initial examination and laboratory evaluation has no apparent cause. *Aseptic meningitis* was defined by a neonate with fever, irritability, poor feeding, vomiting, or bulging fontanelle with pleocytosis >30 cells/mm^3^ in cerebrospinal fluid (CSF), with negative CSF culture for bacteria. *Encephalitis* was defined as a clinical diagnosis by the attending neurologist. It may be accompanied or not by CSF pleocytosis. Electroencephalogram and MRI compatible support the diagnosis but were not considered essential to it, and a clinical criterion of the attending neonatologist was considered enough for inclusion. *Meningoencephalitis* shares data from the two latter ones. *Clinical sepsis* is defined as an infant with an unwell appearance, with alteration of the paediatric assessment triangle (appearance, respiratory and circulatory items). Exclusion criteria were absence of consent or insufficient clinical sample.

From January to December 2013, a total of 84 individual specimens were collected from 84 children up to the age of 1 month, who met the clinical inclusion criteria and were included in the study. Samples were 71 CSFs (85 %), 12 sera (14 %) and 1 throat swab (1 %). Mean age of the children was 13 + 10 days and 46 of them were males (55 %).

### Viral detection and typing

Enterovirus (EV) and herpes simplex virus (HSV-1 and 2) and varicella zoster virus (VZV) screening was performed in the microbiology laboratory from the hospital where the patients were admitted or in the National Centre for Microbiology (CNM), Enterovirus Laboratory, using commercial molecular methods (Xpert EV, Cepheid, CA, USA) or “in-house” RT-PCRs as previously described [8, 21].

HPeV was tested at the CNM in those specimens negative for EV, HSV and VZV, using a real-time RT-PCR designed in the 5-NCR of the genome [7]. Molecular typing of detected EV and HPeV was carried out by 3′-VP1 or VP3/VP1 amplification, respectively, and sequencing [6, 12].

### Statistical analysis

Clinical and laboratory characteristics of the HPeV-positive patients were compared with those of the children infected with EV. A descriptive analysis was performed, expressing qualitative variables as proportions and quantitative variables as mean and standard deviation or median and interquartile range when appropriate. The chi-square test was used to compare groups. A difference with *p* value <0.05 was considered to be significant.

## Results

### Baseline data and frequency of EV and HPeV infections

**Fig. 1:**
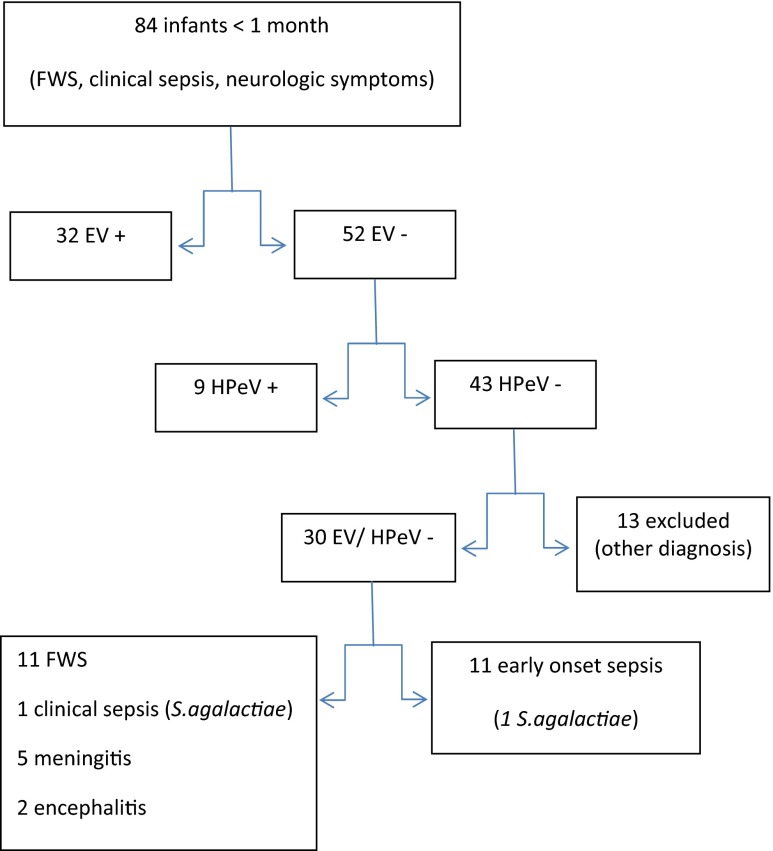
Flow chart of enrolled and tested patients

Out of 84 infants included in the study, 32 were positive for EV (38 %). No HSV-1 and 2 and VZV were detected. The 52 negative specimens (47 CSFs, 4 sera and 1 throat swab) were tested for HPeV, with positive detection in 9 (17 %) of them. Overall, HPeV detection frequency was 11 %. During the clinical course, other diagnosis were made (urinary infection, bronchiolitis, gastroenteritis or non-infectious neurologic disease) for 13 of the infants, and those were finally excluded from the epidemiological and clinical data analysis; all were EV and HPeV negative (Fig. 1).

Viral detections were not found in any patient with suspected early-onset sepsis (11 newborns). In addition, four patients had bacteria positive blood culture, one *Staphylococcus epidermidis* (contaminated culture), two *Streptococcus agalactiae*, and one had a coinfection of *Pasteurella multocida* in blood and echovirus 9 in CSF. It was the only detected virus-bacteria coinfection [1].

### Epidemiological and clinical associations of HPeV infections

**Fig. 2:**
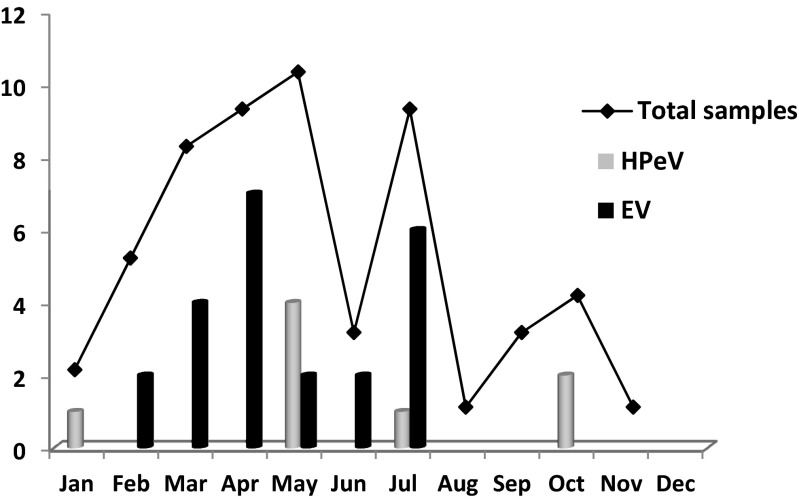
Distribution by month of the enterovirus (*EV*)- and parechovirus (*HPeV*)-positive samples detected in this study

HPeV-positive samples were from four males and five females aged between 6 and 31 days (mean age, 17.6 days). Regarding monthly distribution, most of the HPeV infections (67 %) were detected between April and July, with another peak in October (22 %) (Fig. 2). All viruses were successfully characterized. Eight strains were HPeV-3 and the other was HPeV-5. All but one HPeV-positive specimens were found at CSFs. One of the HPeV-3 was detected in a serum sample. Clinically, six cases were diagnosed as FWS, two as clinical sepsis and one as encephalitis (Table 1). Fever was present in all cases (100 %), 38.6 ± 0.4 °C and lasted a mean of 1.3 ± 1.1 days. Although all except one of the HPeV were detected in CSF, none of the positive cases had cells in the range for pleocytosis in children <1 month of age (up to 30 cells/mm^3^). Protein and glucose were also normal in CSF. Leucocytes in blood were 8500 ± 5696 cells/mm^3^. C-reactive protein and procalcitonine were normal in all patients. Four of the nine HPeV-infected patients (44 %) were admitted to the neonatal intensive care unit (NICU). One of them, diagnosed with encephalitis, had seizures. None of them needed mechanical ventilation, and all experienced total recovery except the infant with encephalitis who had persistent seizures. All patients received antibiotics until the bacterial aetiology could be ruled out.

**Table 1:** Clinical characteristics of infants with human enterovirus (EV) and parechovirus (HPeV) infections

Clinical feature	EV	HPeV	*p*
	(*n* = 32)	(*n* = 9)	
Male	17 (53 %)	4 (44 %)	NS
Age average (days)	17.2+ 7.9	17.6+ 9	NS
Temperature >37.9 °C	31 (97 %)	9 (100 %)	NS
Highest temperature	38.5 ± 0.6	38.6 ± 0.4	NS
Fever duration (days)	1.6 ± 1.1	1.3 ± 1.1	NS
Rash	4 (12.5 %)	1 (11 %)	NS
Antibiotic treatment	23 (72 %)	9 (100 %)	NS
NICU admission	3 (9 %)	4 (44 %)	0.001*
			OR = 5.2(CI:1.9–14)
Leucocytes (cells/mm^3^)	10147 + 4693	8500 + 5696	NS
Serum CRP (mg/L)	10.8 + 27	2.6 + 3.9	NS
Procalcitonine (ng/mL)	0.8 + 2.7	0.2 + 0.09	NS
CSF cells/mm^3^	250 + 415^a^	5.7 + 7	0.015*
			OR = 1.6(CI:1.1–2.1)
CSF proteins (mg/dL)	104 ± 117^a^	49 ± 17	0.030*
CSF glucose (mg/dL)	53 + 10^a^	50 + 8	NS
Diagnosis			
Fever without source	12 (37.5 %)	6 (67 %)	NS
Clinical sepsis	1 (3 %)	2 (22 %)	NS
Meningitis	19 (59.4 %)	0	0.001*
Encephalitis	0	1 (11 %)	NS

Quantitative variables are expressed as mean and standard deviation

^a^Excluded a patient coinfected with EV and *Pasteurella multocida*

*NS* not significant, *NICU* neonatal intensive care unit, *CSF* cerebrospinal fluid, *CRP* C-reactive protein, *OR* odds ratio, *CI* confidence interval

*p values <0.05

### Comparison between EV and HPeV infections

Similar to HPeV infections, 66 % of the total of EV-positive samples were detected between April and July (Fig. 2). Twenty-nine of the 32 EV (91 %) were successfully characterized. All serotypes found belonged to EV species B. Echovirus (E)-18 was the most frequently detected type (7/29, 24 %), followed by E-3 and E-5 (4/29, 14 % each) and coxsackievirus (CV)-B3 (3/29, 10 %). Other types also detected in minor proportion were E-11, E-30, E-6, E-9, E-7, E-17 and CV-B2. The diagnoses for EV-infected patients were meningitis in 19 cases (59.4 %), FWS in 12 cases (37.5 %), and clinical sepsis in 1 case (3 %). Clinical data of the infants are shown in Table 1.

When clinical characteristics of EV-infected patients were compared with those of HPeV-positive children, we observed significant differences in the pleocytosis in CSF (250 ± 415 cells/mm^3^) *p* = 0.015. The odds ratio (OR) for pleocytosis and EV infection was OR = 1.5 (confidence interval CI:1.1–2.1). CSF protein were also higher in EV group (104 ± 117 vs. 49 ± 17 mg/dL, *p* = 0.03). Furthermore, meningitis diagnosis was significantly more frequent in EV-positive patients than in HPeV-infected children (19/32 vs. 0/9, *p* < 0.001). Out of the 32 EV-positive patients, 23 (72 %) received antibiotic treatment. Only three EV-positive patients (9 %) needed admission to the NICU and no sequelae were described in this group. However, HPeV infection was a risk factor for admission to the NICU (OR = 5.2 (CI: 1.9–14)) (3/32 vs. 4/9, *p* < 0.001).

## Discussion

We present a prospective, multicentre study performed during 1 year in Spanish infants up to 1 month of age with suspected sepsis or meningo-encephalitis or fever without source. EV was detected in 38 % of the patients and HPeV in 11 %. With respect to EV infections, other authors found EV incidences in neonates between 9.5 and 24 % [2, 16, 18, 26]. The high detection frequency observed in our series might be due to the study being performed in clinically ill infants with a high suspicion of viral infection and not as active surveillance. Besides, not all EV-negative samples collected by the hospitals could be included in the final study, due to absence of available samples or signed consent forms.

On the other hand, few HPeV epidemiological studies focused on newborns (infants <30 days) have been published. Most of them were performed in children (<18 years), and the presence of the virus in CSF varies from 2.3 to 7 %, whereas in the general population the percentage decreases to 0.5–0.8 % [9–11, 23]. As far as we know, there is only one study from Italy in which 7 (11.6 %) cases of EV and 3 (5 %) cases of HPeV infections were detected out of 60 neonates with suspected sepsis or neurological infection [18]. Our higher HPeV prevalence in the same type of patients (11 %) might be due to the fact that the Italian study [18] was conducted at the NICU, reducing the study population and leaving out the analysis of children who do not require admission to the NICU (56 % in our series).

In most of the previous studies, HPeV-3 infections have been described as clearly associated with central nervous system infections and sepsis-like illness in neonates and young infants [4, 15, 22]. Our data supported these findings, as HPeV-3 was also the predominant genotype identified, representing 89 % of the total HPeV detected. Only one strain was identified as HPeV-5, which is an infrequent type reported in the literature [9–11, 18, 23]. In this study, this type was detected in a case with FWS, so we cannot conclude that HPeV type 5 has a proved association with neurological or systemic diseases in newborns.

Regarding seasonal distribution, in Europe, HPeV infections are more common in spring and summer, similar to the EV infections [9–11, 23]. In our series, there is also a predominance of HPeV detection in spring and summer, although we have only 1 year of enrollment period. Besides, HPeV has been described to present in biannual cycles in Europe [9, 11, 15]. Previous data in Spain (not published) are consistent with those findings as the detection frequency was significantly higher in 2011 and 2013 than in 2012, but further longer surveillance studies are necessary to confirm the seasonality and periodicity of the HPeV-3 infections. This circulation pattern might also explain the high HPeV incidence found in this study, in comparison with the previously mentioned Italian one [18].

Recently, Sharp et al [23], in Kansas, found that up to 17 % of the CSF-positive samples of children (<18 years) tested between June and October 2009 were positive for HPeV, with HPeV-3 being the predominant type. Clinically, they described HPeV infections with irritability and longer fever, lower peripheral leucocytes, absence of pleocytosis, lower CSF protein and higher CSF glucose compared to EV infections. In our series, some of these clinical characteristics were also observed as HPeV-infected patients had lower blood leucocytes, CSF protein and CSF cells values than those with EV infection. Furthermore, several studies including the American one mentioned above reported that admission to intensive care units were more frequent in HPeV-positive cases than in those infected by EV, but the prognosis was generally good [9, 22, 23]. We observed the same in our series. The higher proportion of HPeV-infected children who required admission to NICU was probably determined by the low age in an infant with an unwell appearance rather than by the clinical severity, as there was only one patient with encephalitis who had sequelae. In the literature, however, some fatal cases were described [19, 20], encephalitis being the major complication that caused sequelae and deaths.

Another recent study reported that between 50 and 100 % of HPeV-positive cases had been associated with erythematous rash on the extremities, especially palms and soles, not always present in the first hours but during evolution [24]. This dermatologic manifestation would be very suspicious data of HPeV infection in neonates and very young infants with febrile syndrome. However, these data are not consistent in the literature; the rash might have gone unnoticed or showed a different prevalence depending on the area. In the present study, only 12.5 % of patients showed rash.

In summary, HPeV-3 infections should be suspected in infants younger than 1 month, with fever but without leucocytosis and pleocytosis in CSF. In these cases, HPeV screening in CSF or blood should be incorporated in the routine viral diagnosis to prevent unnecessary antibiotic treatment and prolonged hospitalization. The low number of samples included in this study was a limitation that affects the incidence data of the EV and HPeV infections, but not the results obtained in relation to the clinical and epidemiological differences found between both infections. Further studies could provide more information about the burden of HPeV infections in newborns.

## Abbreviations

CSF: Cerbroespinal fluid
EV: Enterovirus
HPeV: Human parechovirus
FWS: Fever without source
NICU: Neonatal intensive care unit
